# Pharmacovigilance and Pharmacoepidemiology as a Guarantee of Patient Safety: The Role of the Clinical Pharmacologist

**DOI:** 10.3390/jcm11123552

**Published:** 2022-06-20

**Authors:** Giada Crescioli, Roberto Bonaiuti, Renato Corradetti, Guido Mannaioni, Alfredo Vannacci, Niccolò Lombardi

**Affiliations:** 1 Pharmacovigilance and Pharmacoepidemiology Research Unit, Section of Pharmacology and Toxicology, Department of Neurosciences, Psychology, Drug Research and Child Health, University of Florence, 50139 Florence, Italy; roberto.bonaiuti@unifi.it (R.B.); renato.corradetti@unifi.it (R.C.); guido.mannaioni@unifi.it (G.M.); 2 Tuscan Regional Centre of Pharmacovigilance, 50122 Florence, Italy; 3 Pharmacology Unit, Careggi University Hospital, 50134 Florence, Italy; 4 Medical Toxicology Unit and Poison Control Centre, Careggi University Hospital, 50134 Florence, Italy

## 1. Background

Recent years, particularly the COVID-19 pandemic, can be considered a turning point for pharmacovigilance and pharmacoepidemiology in terms of their role in drug safety and drug utilisation monitoring in clinical practice [1]. Researchers operating in the fields of pharmacovigilance and pharmacoepidemiology have extensive knowledge about approved medications, many of which have been or are currently undergoing clinical trials for repurposing. In this context, clinical pharmacologists’ knowledge can be used and translated to optimise dosing and treatment regimens and to assess the relationship between active compound exposure and adverse drug reactions (ADR) or adverse events following immunisation (AEFI), with the crucial aims of optimising drugs or vaccines’ efficacy and ensuring patients’ safety.

## 2. Pharmacovigilance

Adverse drug reactions represent a relevant clinical issue, causing each year a significant number of medical consultations, emergency department (ED) visits, and/or patients’ hospitalisations [2], including an increase in the length of patients’ hospital stay. ADRs are considerable medical occurrence, not only from a clinical point of view, but they also represent an economic burden, since they can be involved in the death of several thousand patients each year, accounting up for a not negligible percentage of a hospital’s budget [3]. This scenario is even more complex if we consider that many ADRs are predictable and therefore preventable.

Drug and vaccine safety surveillance is a continuous process, which includes all the phases of the life cycle of a drug/vaccine. Additionally, also surveillance of the safety of products belonging to complementary and alternative medicine (CAM), defined as phytovigilance [4], contributes to guaranteeing the safety of patients.

During the drug development process, safety is investigated in different preclinical and clinical phases. Although drug and vaccine safety evaluation is very rigorous and highly regulated, randomised clinical trials (RCTs) have several limitations, which include limited numbers of patients, strict eligibility criteria, and limited duration. These intrinsic characteristics of RCTs make their results far from representing the real-world population, thus not allowing an exhaustive assessment of the safety profile of drugs and vaccines. Consequently, post-marketing surveillance, also known as pharmacovigilance (during phase IV of drug development), plays a key role in better defining drugs’ and vaccines’ safety profiles in clinical practice, overcoming the gap of evidence derived from the pre-marketing phases [5,6]. These aspects are even more relevant if we consider that most CAM products (i.e., dietary supplements, herbal supplements, traditional Chinese medicine products, homoeopathic products, etc.) are placed on the market without first being tested on humans, even less in frailer subgroups [6].

In the last decades, spontaneous reporting of suspected ADRs, AEFIs, and adverse events associated with CAM products, has represented the starting point and the milestone to build the modern pharmacovigilance system which is currently operating, although with differences between countries, all over the world under the coordination of the World Health Organisation Uppsala Monitoring Centre. More recently, new methodological approaches have become necessary in the field of pharmacovigilance, to try to overcome the limits of spontaneous reporting, particularly the underreporting [2].

An important example is represented by active pharmacovigilance projects, aimed at estimating the impact of ADRs, AEFIs, and adverse events associated with CAM products through continuous pre-organized activities in specific clinical settings (i.e., ED, hospital ward, nursing home, etc.) [2,5] or through the analysis of local or national administrative healthcare databases (i.e., dispensed drugs, emergency department records, hospital discharge records, exemption codes, etc.) [7].

In the frame of ED, active pharmacovigilance represents a valuable methodology, allowing healthcare professionals and researchers to detect, collect and characterise the clinical burden of ADRs [6], AEFIs [8], and adverse events associated with CAM products [9,10] in outpatients. Furthermore, active pharmacovigilance may help to recognise risk factors associated with adverse reactions among specific patient populations, such as the elderly (age ≥ 65 years) [11], women [7], including those in pregnancy or breastfeeding [12,13], children [14], subjects exposed to polypharmacy, or patients affected by substance use disorder [15]. In this framework, an active pharmacovigilance approach can help to early recognise and prevent adverse reactions, minimising their clinical, economic and social impact.

In recent years, new technologies are also making an important contribution to active pharmacovigilance. In particular, machine learning, deep learning, and natural language processing approaches can be used to detect adverse reactions from unconventional data sources, e.g., social media [16,17], and to discover safety signals, underlying some adverse reactions not yet reported [18]. Moreover, safety data collected from healthcare social networks and forums, general social networking, and search logs can be processed by big data sentiment analysis algorithms to provide a more comprehensive picture of the public opinion, experiences, and sentiments about drugs and vaccines [19], and can be used to create effective public health campaigns on drugs/vaccines safety, diseases prevention or to fight vaccine hesitancy [20].

## 3. Pharmacoepidemiology

Pharmacoepidemiology refers to the study of interactions between drugs, vaccines, or CAM products and populations. In particular, it can be defined as the study of the utilisation, therapeutic effects, and risks of different health products through several epidemiological approaches. Even though RCTs may be preferred for evaluating the efficacy of a drug or a vaccine, the principal aim of the pharmacoepidemiological method is to estimate drug or vaccine effect in real life (effectiveness), avoiding, as much as possible, any modification caused by the study itself (i.e., presence of biases). From a practical point of view, pharmacoepidemiology relates to descriptive methodologies (i.e., describing the use of a drug in a specific demographic or clinical setting) [21], as well as to etiologic methodologies (i.e., estimating the association between drug exposure and a specific clinical outcome) [22]. Researchers operating in the field of pharmacovigilance, first the clinical pharmacologist, can use pharmacoepidemiological studies to identify unexpected or rare safety issues and detect changes in frequency of expected ADRs/AEFIs, making possible a continuous monitoring of the benefit risk ratio of a drug, vaccine or CAM product in the real-world setting [5].

New evidence concerning drug safety obtained through pharmacoepidemiological approaches is relevant for pharmaceutical industries because they can use this information to submit amendments to the approved indications for their products. In fact, pharmacoepidemiology allows industries to demonstrate the safety of their products, especially compared to others (comparative observational studies). Furthermore, it is also fundamental for regulatory agencies, for physicians, and patients using health products. In this context, there is a clear need for high-quality epidemiological research in the post-marketing phase for the timely identification of any significant safety concerns that arise when a drug, vaccine, or CAM product is used in the real-world “uncontrolled” setting. All this is mandatory to protect the health and quality of life of patients.

Over the past decades, more data has become increasingly available due to the constant growth of administrative healthcare databases, in particular those reporting data on prescription drugs. This circumstance has enhanced the improvement of pharmacoepidemiology, a dynamic research field that has undergone a more rapid development than many other research areas of pharmacology and clinical pharmacology. Evidence proliferation in modern society will continue, and population-based observational studies (i.e., cohort and case control studies) aimed at assessing the effectiveness (defined as the extent to which a drug achieves its intended effect in the usual clinical setting) and safety of drugs, vaccines, and CAM products will be increasingly requested by industry, regulatory agencies, payers, healthcare professionals, patients, and caregivers.

The availability of electronic healthcare databases will enable researchers operating in the fields of pharmacovigilance and pharmacoepidemiology to identify a growing number of cases in which effectiveness does not match efficacy. This will challenge the actions of all concerned stakeholders. For these reasons, pharmacoepidemiological studies will also be increasingly requested by reimbursement agencies and other payers to assess the value of health products used in the general population. In conclusion, it is essential that the increasing amount of data collected to monitor the utilisation, effectiveness, and safety of new drugs, vaccines, and CAM products will be used to improve clinical decision-making. In this complex scenario, the clinical pharmacologist will certainly have to play an important role, in concert with the other actors involved in the post-marketing setting.

## 4. The Role of the Clinical Pharmacologist

In the real-world setting, there are two different actors in the issue of ADRs: the healthcare professional (i.e., medical doctor, pharmacist, nurse, etc.) and the citizen/patient. While they are directly involved, in cooperation with universities, regulatory authorities, and drug manufacturers, the figure of the clinical pharmacologist (with a medicine or pharmacy single-cycle degree background) has also a pivotal role in the management of adverse reactions. However, his/her role is still often underestimated and underused [23].

Generally, the clinical pharmacologist has a dual background, in biomedical science and in pharmacology. Furthermore, a clinical pharmacologist will often be trained in biostatistics and pharmacoepidemiology. For example, in Italy, to become a clinical pharmacologist, a single-cycle master’s degree in healthcare (i.e., medicine or pharmacy), followed by a doctorate and/or a specialisation in clinical pharmacology and toxicology is needed. Clinical pharmacologists operate in universities, industry, regulatory authorities, and hospitals. In fact, their activities comprehend teaching in biomedical schools, research in the public or industry fields, regulatory affairs, and hospital activities.

In Italy, the main healthcare professionals that are involved in pharmacovigilance are hospital pharmacists. However, while the hospital pharmacist is directly involved in the management of pharmacovigilance report forms, including all activities related to the proper functioning of the national pharmacovigilance system, clinical pharmacologists have a unique insight into the possible mechanism of the putative adverse reaction, the underlying or concomitant diseases, and the possibility of drug–drug, drug–CAM or drug–disease interactions [1,24]. Thus, more recently, hospital pharmacists have been supported by one or more clinical pharmacologists, and their cooperation at both hospital and territorial level enhance the knowledge of drug safety in the pre- and post-marketing settings. Moreover, the clinical pharmacologist may enhance the quality of information and may help in the interpretation of data collected during adverse events reporting. This could be of utmost importance for modern projects of active pharmacovigilance when the clinical pharmacologist can play the role of a trained monitor.

At the individual patient level, the clinical pharmacologist could be involved in the resolution of several drug-related issues, which can vary from some advice regarding drug administration, to the choice of a drug in specific population subgroups, such as pregnant women. Indications in adverse reactions management can be based on literature evidence or on knowledge of drugs’ pharmacokinetics and pharmacodynamics, or they may be part of a more proactive approach. In proactive and preventive approaches, the clinical pharmacologist could take part in the resolution/prevention of adverse reactions, cooperating with other healthcare professionals to improve the appropriateness of use of drugs and CAM products and to increase personalised medicine [25]. Moreover, new technologies for adverse reactions monitoring could be improved by clinical pharmacologists’ knowledge. These new applications and tools can be designed taking into account many aspects of pharmacology, pharmacoepidemiology, and pharmacological use in real clinical practice in which the clinical pharmacologist is an expert.

During the SARS-CoV-2 pandemic, great attention was paid to the risk associated with the involuntary intoxication and to the need for correct information concerning the in-home utilisation of several medical and non-medicinal products (i.e., cleaners and disinfectants, medical devices, etc.), thus representing a further field of action in which the clinical pharmacologist can guarantee a fundamental contribution [26]. Actually, the role of clinical pharmacologists, particularly those working in a poison control center, is valuable in the identification and management of exposures and suspected intoxications in the general population or in specific subgroups [27].

## 5. Future Perspectives

Real-world data are crucial to further establish the safety profile of pharmacological treatments in the general population [28]. As a next step, real-world data from electronic healthcare databases may be used in pharmacovigilance and pharmacoepidemiology to monitor drug utilisation patterns, as well as the effectiveness and safety of drugs in large populations. Big data and machine learning analysis technologies could be used to extract and aggregate pharmacovigilance data from different and unrelated sources (i.e., health insurance companies, academic institutions, healthcare systems, etc.) and associate relevant information on drug safety using artificial intelligence tools [29]. All these approaches will be particularly useful in the near future for the monitoring of COVID-19 vaccines’ safety. Moreover, among new technologies, gamification is another promising technology with several potential uses in drug safety. Gamification applications transfer gaming dynamics to the “serious context” of pharmacological therapies, for example, to educate health professionals [30] and patients [31] on the correct management of chronic diseases or to improve adherence to drug treatments thus reducing adverse events [32]. In this context, “digital therapeutics” (algorithms and software) can represent an innovative approach through which the clinical pharmacologist can improve the management and safety of pharmacological and/or integrative treatments [33].

As the amount of data available from randomised controlled trials and pharmacoepidemiological studies will increase over the coming years, the characteristics of adverse events related to medicines, vaccines or CAMs will become clearer and the central role of the clinical pharmacologist should become even more relevant. Managing adverse events should be a routine activity for clinical pharmacologists. This management takes advantage of all the skills of the clinical pharmacologist to explore individual cases and the mechanisms of adverse events. With their knowledge in pharmacovigilance and pharmacoepidemiology, clinical pharmacologists may play a key role by bringing various healthcare professionals together in a proactive discussion/collaboration aimed at improving the safety of pharmacological and/or integrative treatments in clinical practice. 
